# Commentary: Totality of the Evidence Suggests Prenatal Cannabis Exposure Does Not Lead to Cognitive Impairments: A Systematic and Critical Review

**DOI:** 10.3389/fpsyg.2021.651064

**Published:** 2021-03-04

**Authors:** Lynn T. Singer, Barbara A. Lewis, Julia S. Noland

**Affiliations:** ^1^Pediatrics, Psychiatry and Psychological Sciences, School of Medicine, Case Western Reserve University, Cleveland, OH, United States; ^2^Department of Psychological Sciences, Case Western Reserve University, Cleveland, GA, United States; ^3^Department of Psychology and Human Development, Vanderbilt University, Nashville, TN, United States

**Keywords:** cognitive, prenatal, cannabis exposure, neurotoxicology, developmental

Data from the National Survey on Drug Abuse and Health indicate that cannabis use more than doubled among pregnant women in the United States from 2002 to 2017, (Volkow et al., 2019) raising the importance of understanding the effects of prenatal cannabis exposure on infant development.

A recent review by Torres et al. (2020) stated that it “provides a critical review of the impact of prenatal cannabis exposure (PCE) on multiple domains of cognitive functioning.” The authors examined 1,001 statistical comparisons between PCE vs. non-exposed samples, resulting in poorer performance on <3.5% vs. better in <1%. The scores of PCE groups fell within normal limits when compared to education adjusted norms. They concluded that current evidence does not suggest that PCE is associated with clinically significant cognitive impairments, and that their article could have important public health policy implications.

We have significant concerns about this paper in methodology and interpretation of findings. The authors erroneously included nine papers (Singer et al., 1999, 2002, 2005, 2008; Noland et al., 2003a,b, 2005; Lewis et al., 2004, 2011) as the Case Western Reserve University cohort. Our cohort was composed for a longitudinal study to assess developmental sequelae of prenatal cocaine exposure, not cannabis, and every description of our studies describe them as such. Recruited women were heavy crack-cocaine users, also using alcohol, tobacco, cannabis, and other drugs, with the latter substances treated as covariates. Associations of other substances with outcomes were reported only for heuristic purposes.

An accurate interpretation of the relationship of PCE to cognitive outcomes from our studies would be that, in a group of cocaine, polydrug exposed children, no contribution of marijuana to cognitive outcome could be determined. Statistically, after entering cocaine, alcohol, and tobacco in the model, little variance was left to be explained by cannabis.

Any critical review would have eliminated our cohort as it does not provide, nor was intended to provide, an assessment of the contribution of PCE to cognitive outcomes, based on the stated research design and aims to assess prenatal cocaine exposure and developmental outcomes.

Ten additional studies included in the review were also designed to assess the effects of prenatal cocaine exposure, not PCE, including (Frank et al., 2005; Hurt et al., 2005, 2009; Beeghly et al., 2006; Morrow et al., 2006; Mayes et al., 2007; Bennett et al., 2008; Carmody et al., 2011; Rose-Jacobs et al., 2011, 2012). Each study makes clear that their focus is prenatal cocaine exposure, not PCE. Their inclusion inaccurately inflates the importance of the absence of evidence since PCE was a covariate and not the focus of study design.

The authors note that “Cognitive scores (were) not reported.” For our studies, cognitive scores related to cannabis were not reported as they were not valid within the context of the design of the studies.

Only the Ottawa and Pittsburgh studies reviewed were designed to address prenatal cannabis exposure. Another large study of PCE, the Generation R Study (El Marroun et al., 2009) was inexplicably not included.

Also, the review noted that the clinical significance of differences between PCE and non-exposed groups was not examined, since scores were not compared with normative data for age and education. However, standard scores reported for outcomes already provide the group's relative standing based on norms for the reference population. Using a control group of similar age, race, and socioeconomic status, as the Ottawa and Pittsburgh studies have done, actually provides a more refined test of differences since it controls, either through design or statistically, confounding factors in addition to age and education (Singer et al., 2020). Those confounders are not accounted for in the normative samples and results would be more inaccurate as to the differences in cognition. The clinical significance of differences on a population level can be considered through the effect size. The authors' assertion that, because cognitive scores reported were within normal limits, there is no clinical significance, ignores also population level significance.

We have addressed this issue with the author previously (Singer, 2020), noting that standardized test scores present normative comparisons but do not evaluate population differences (Lester et al., 1998).

There are many factual inaccuracies, inconsistencies, and misinterpretations in the review. As examples, the authors did not differentiate between experimental tasks (Singer et al., 1999; Noland et al., 2005) and standardized tests, citing all studies as not reporting cognitive “scores.”

The papers by Lewis et al. (2004, 2011) are described as “did not control for other drugs,” which is inaccurate.

The paper by Singer et al. (2008) is described as “In an attempt to minimize the impact of other drug use, Singer et al. (2008) conducted a study in which the cognitive function of 9-year-old children with prenatal cannabis exposure…was examined.” As noted, our study compared prenatal cocaine exposed with non-exposed children, as described in the study's aims and subject composition. If the relationship of PCE to cognitive outcomes were our intent, the use of a comparison group of heavy crack-cocaine exposed children would have been inappropriate.

Finally, we have strong reservations about the validity of the idiosyncratic methodology of this review. The appropriate method to address the authors' stated concerns about the differences between studies on multiple levels is a meta-analysis and their reasons for not conducting one are not convincing.

In summary, we have major concerns about the inclusion of the Case Western Reserve University studies in this review as they were designed and described as studies of prenatal cocaine exposure and have never been included in any reviews of prenatal cannabis exposure. The misrepresentation of our cohort, as well as others, as addressing PCE by their inclusion is misleading and can have harmful public health effects by suggesting that they support evidence that PCE alone is not associated with clinically significant cognitive functioning impairments (Jacobson and Jacobson, 2005). Indeed, such conclusions are already being falsely promoted in the popular literature based on this critique (Burns, 2020).

## Author Contributions

All authors listed have made a substantial, direct and intellectual contribution to the work, and approved it for publication.

## Conflict of Interest

The authors declare that the research was conducted in the absence of any commercial or financial relationships that could be construed as a potential conflict of interest.
